# Single Parameter Estimation Approach for Robust Estimation of SIR Model With Limited and Noisy Data: The Case for COVID-19

**DOI:** 10.1017/dmp.2020.220

**Published:** 2020-06-25

**Authors:** Kerem Senel, Mesut Ozdinc, Selcen Ozturkcan

**Affiliations:** Faculty of Health Sciences, Istanbul University - Cerrahpasa, Istanbul, Turkey; School of Economics and Business, Åbo Akademi University, Turku, Finland; Department of Statistics, Mimar Sinan FA University, Istanbul, Turkey; School of Business and Economics, Linnaeus University, Kalmar, Sweden; Sabanci Business School, Sabanci University, Istanbul, Turkey

**Keywords:** coronavirus, COVID-19, epidemic models, robust estimation, SIR

## Abstract

**Objective:**

The susceptible-infected-removed (SIR) model and its variants are widely used to predict the progress of coronavirus disease 2019 (COVID-19) worldwide, despite their rather simplistic nature. Nevertheless, robust estimation of the SIR model presents a significant challenge, particularly with limited and possibly noisy data in the initial phase of the pandemic.

**Methods:**

The K-means algorithm is used to perform a cluster analysis of the top 10 countries with the highest number of COVID-19 cases, to observe if there are any significant differences among countries in terms of robustness.

**Results:**

As a result of model variation tests, the robustness of parameter estimates is found to be particularly problematic in developing countries. The incompatibility of parameter estimates with the observed characteristics of COVID-19 is another potential problem. Hence, a series of research questions are visited.

**Conclusions:**

We propose a Single Parameter Estimation (SPE) approach to circumvent these potential problems if the basic SIR is the model of choice, and we check the robustness of this new approach by model variation and structured permutation tests. Dissemination of quality predictions is critical for policy- and decision-makers in shedding light on the next phases of the pandemic.

Coronavirus disease 2019 (COVID-19) is recognized as the worst pandemic in modern times in terms of both mortality and infectiousness since the flu pandemic of the early 20th century, ie, the so-called Spanish flu. The first case being reported in the Republic of China on December 8, 2019,^1^ COVID-19 spread quickly into other countries and continents, which led to its classification as “pandemic” by the World Health Organization (WHO) on March 11, 2020.^2^

The susceptible-infected-removed (SIR) model is widely used to predict the progress of COVID-19 in many countries,^3-10^ despite its rather simplistic nature, such as its underlying assumptions regarding the homogeneity of the population. It is a basic deterministic compartmental model that simplifies the mathematical modeling of infectious diseases. Its origins date to the seminal work by Kermack and McKendrick in the early 20th century.^11^ The model involves many variants, such as the SIRD model,^12^ the MSIR model,^13^ the SEIR model,^14^ the MSEIR model,^15^ and the SIR-A model.^16^

Although deterministic models such as the SIR are simpler than stochastic or agent-based simulation models, a deterministic model may be preferred in the case of COVID-19. This is especially the case for developing and underdeveloped countries where quality and detailed data required by more sophisticated models may be hard or even impossible to collect. Stochastic models are better suited for smaller populations, whereas agent-based simulation models require numerous parameters to be estimated, and they are also more challenging to interpret and perform sensitivity analysis on.^17^

On the other hand, the robust estimation of even the most basic SIR model parameters is a significant challenge, especially with limited and potentially noisy data in the initial phases of the pandemic.^18^ Another problem with parameter estimation is observed on the discrepancy between parameter estimates and actual disease characteristics. These potential problems shadow the reliability of model outputs, which are most needed by decision- and policy-makers in forecasting the progress of the pandemic and taking the necessary measures accordingly.

Our study addresses 4 research questions regarding the basic SIR model: (1) Is it possible to estimate the model parameters simultaneously in a robust manner? (2) What is the impact of time on the degree of robustness? (3) Are there any significant differences between countries in terms of robustness? (4) Is it possible to obtain model parameters that are compatible with actual disease characteristics when model parameters are estimated simultaneously?

Accordingly, we have 4 testable hypotheses corresponding to these research questions: *Hypothesis 1:* Robust estimation of model parameters is not possible if the model parameters are estimated simultaneously. *Hypothesis 2:* Robustness improves with more data as time progresses. *Hypothesis 3:* Robustness is relatively more problematic for developing countries compared with developed countries. *Hypothesis 4:* Simultaneous estimation of model parameters leads to parameter estimates that are not compatible with actual disease characteristics.

This study has 2 primary objectives. We first focus on the problems in the estimation of the basic SIR model parameters and their real-life implications observed throughout the development of COVID-19. Second, we propose a Single Parameter Estimation (SPE) approach that enables us to obtain robust parameter estimates. This approach also helps to bridge the gap between parameter estimates and actual disease characteristics.

It is also imperative to point out that it is more appropriate to use more sophisticated models than the basic SIR model whenever the available data permits. Our proposed approach is not a panacea or a general modeling method for modeling COVID-19 or any other pandemic. It is just a convenient way of obtaining robust parameter estimates if the basic SIR is the model of choice.

## THE SIR MODEL

The SIR model assumes 3 homogeneous compartments that comprise the population. Hence, it may not be appropriate to use this model if the population under consideration is remarkably heterogeneous. A prime example of such heterogeneity is in the United States of America. There is a stark difference between New York and the rest of the country in terms of the impact of COVID-19. As of May 30, 2020, 11.5% of all confirmed cases in the United States are in New York City,^19^ which represents a mere 2.6% of the total population.^20^ This difference is mainly due to population density, which affects the transmission dynamics of the disease.

S, I, and R stand for the number of susceptible, infected, and removed individuals, respectively. Removed individuals are those who either recovered or lost their lives so that they can no longer spread the disease. The SIR model is represented by 3 differential equations (1, 2, and 3) Sthat define the change in these variables with respect to time.(1)
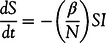

(2)
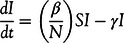

(3)
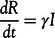



In Equations (1) to (3), *N* is the population, whereas *β* and *γ* are the infection and recovery rates, respectively. In most studies, *N* is assumed to be constant, which is also a reasonable assumption for the case of COVID-19. Hence, *β* and *γ* are the parameters to be estimated.

## ROBUSTNESS OF PARAMETER ESTIMATES

Problems arise when these parameters are estimated simultaneously, particularly with limited and potentially noisy data at the initial phase of the pandemic. We first observed these problems with our own code in R when we estimated the model parameters for successive dates.^8^ The model parameter estimates were not robust from 1 d to the next, and the estimated parameters were not compatible with actual disease dynamics. We observed the same problems in another study that reported the SIR model parameter estimates for successive dates.^10^ Realizing that these problems arise from the lack of sufficient number of data points, we adopted an approach to take *γ* from the literature and estimate *β* only.^8^

For this study, we decided to use the code authored by Batista in MATLAB^10^ instead of our own code in R.^8^ The reason behind this choice is 2-fold. First, the code written by Batista is open to the public, and it has been downloaded 1123 times, with an average 5-star rating of a total of 43 ratings as of May 31, 2020.^21^ Therefore, the code is subject to public and expert scrutiny and more reliable from the viewpoint of an outsider compared with our own code in R. Second, the code was used in a very popular study by the Singapore University of Technology and Design (SUTD) that tried to estimate the ending dates of the COVID-19 for different countries.^22^ The predictions of this study proved to be inaccurate, and we think that this is closely related to the problems associated with the estimation of SIR model parameters. Using the same code by Batista may provide further insight into why these predictions have gone awry. Other than these motivations, there is nothing special behind our choice of code. There is also nothing faulty about the code authored by Batista apart from the universal problems of estimation, which mainly stem from the lack of sufficient and quality data.

Batista authored a function in MATLAB, “fitVirusCV19”, to implement the SIR model,^10^ for which we selected the top 10 countries with the highest number of COVID-19 cases as of May 20, 2020^23^ to apply the SIR model by means of fitVirusCV19. As a model variation test, the estimates of *β* and *γ* and the absolute value of the percent daily changes in parameter estimates are presented in Table 1 for April 21 and 22, 2020.

**TABLE 1:** *β* and *γ* Estimates With % Daily Change Between April 21 and 22, 2020

Country	*β* 04/21/2020	*β* 04/22/2020	abs(%*Δβ*)	*γ* 04/21/2020	*γ* 04/22/2020	abs(%*Δγ*)
Brazil	0.927	0.797	14.0%	0.793	0.663	16.4%
France	0.327	0.320	2.1%	0.163	0.157	3.7%
Germany	0.336	0.330	1.8%	0.160	0.156	2.5%
Iran	2.036	1.528	25.0%	1.930	1.422	26.3%
Italy	0.294	0.297	1.0%	0.157	0.163	3.8%
Russia	0.742	0.433	41.6%	0.579	0.268	53.7%
Spain	0.339	0.332	2.1%	0.161	0.157	2.5%
Turkey	0.331	0.907	174.0%	0.180	0.743	312.8%
United Kingdom	0.349	0.347	0.6%	0.191	0.191	0.0%
United States	0.360	0.350	2.8%	0.188	0.183	2.7%
Mean	0.604	0.564	26.5%	0.450	0.410	42.4%
Median	0.344	0.349	2.5%	0.184	0.187	3.8%

The results support *Hypotheses 1* and *3*. Parameter estimates change significantly from 1 d to the next, and the daily changes are particularly pronounced for developing countries.

The countries can be broadly categorized into 3 groups in terms of the robustness of parameter estimates. For France, Germany, Italy, Spain, the United Kingdom, and the United States, the absolute value of the percent daily change in parameter estimates ranges between 0.6% and 2.8% for *β* and 0.0% and 3.8% for *γ*. For Brazil, Iran, and Russia, the absolute value of the percent daily change in parameter estimates ranges between 14.0% and 41.6% for *β* and 16.4% and 53.7% for *γ*. Turkey stands out as an outlier with very high percent daily changes in both parameter estimates.

**FIGURE 1:**
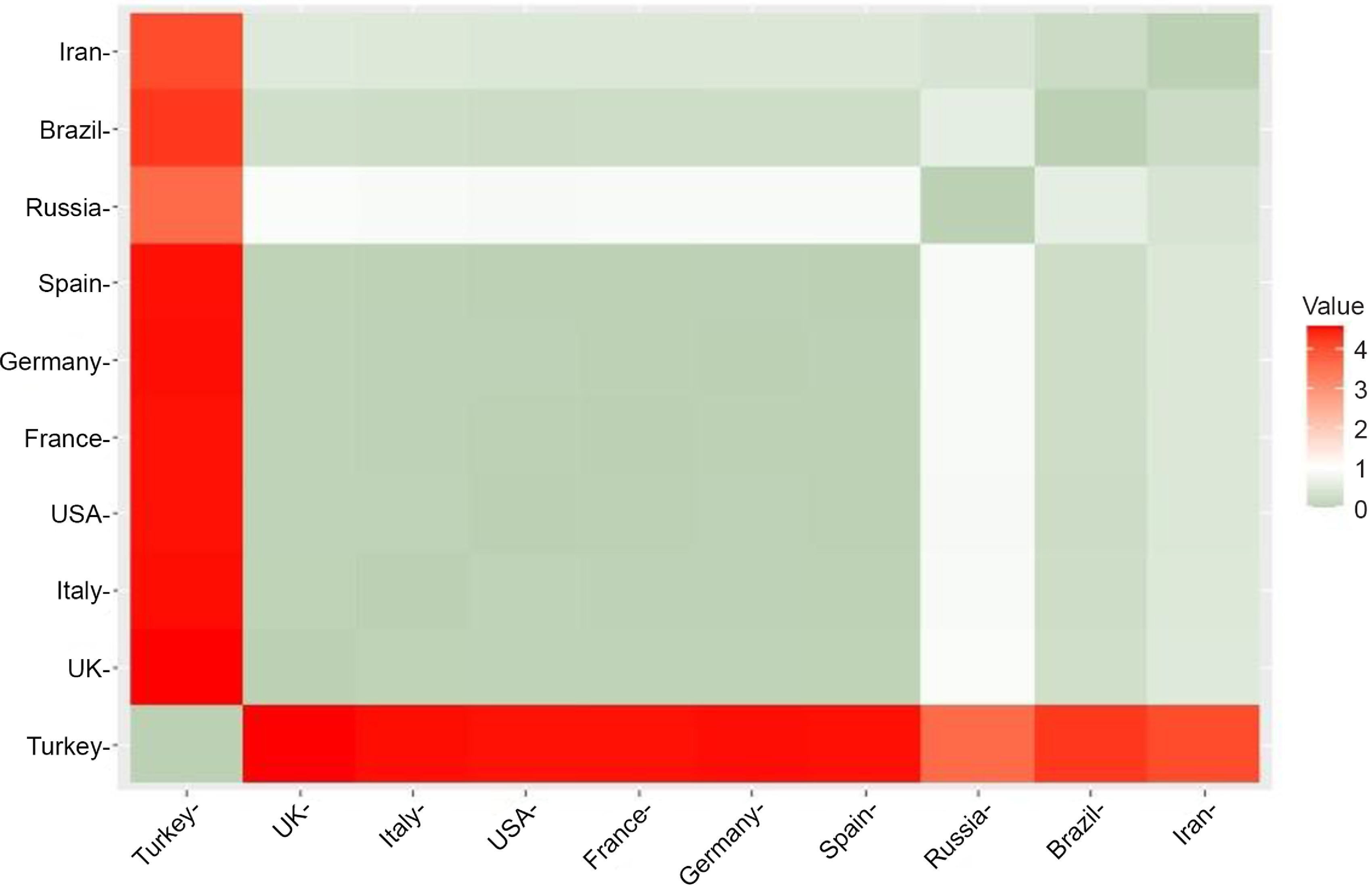
Distance Matrix Calculated From abs(%*Δβ*) and abs(%*Δγ*) for April 21 and 22, 2020.

Figure 1 shows a graphical representation of the distance matrix of countries calculated from abs(%*Δβ*) and abs(%*Δγ*) for April 21 and 22, 2020. If the color of a box is green (smaller distance), it means that the corresponding 2 countries are similar in terms of robustness. A red box, on the other hand, is an indication of greater distance and dissimilarity.

To perform a formal cluster analysis, we used the k-means algorithm. K-means is one of the most popular unsupervised machine learning algorithms to group similar data points into clusters and discover underlying patterns.^24^ The algorithm identifies k number of centroids, ie, the imaginary or real locations representing the centers of the clusters, and then allocates every data point to the nearest cluster. The most common distance metric is the usual Euclidean distance, but it is possible to use other metrics, such as the Manhattan distance, Chebyshev distance, or the Minkowski distance.

**FIGURE 2:**
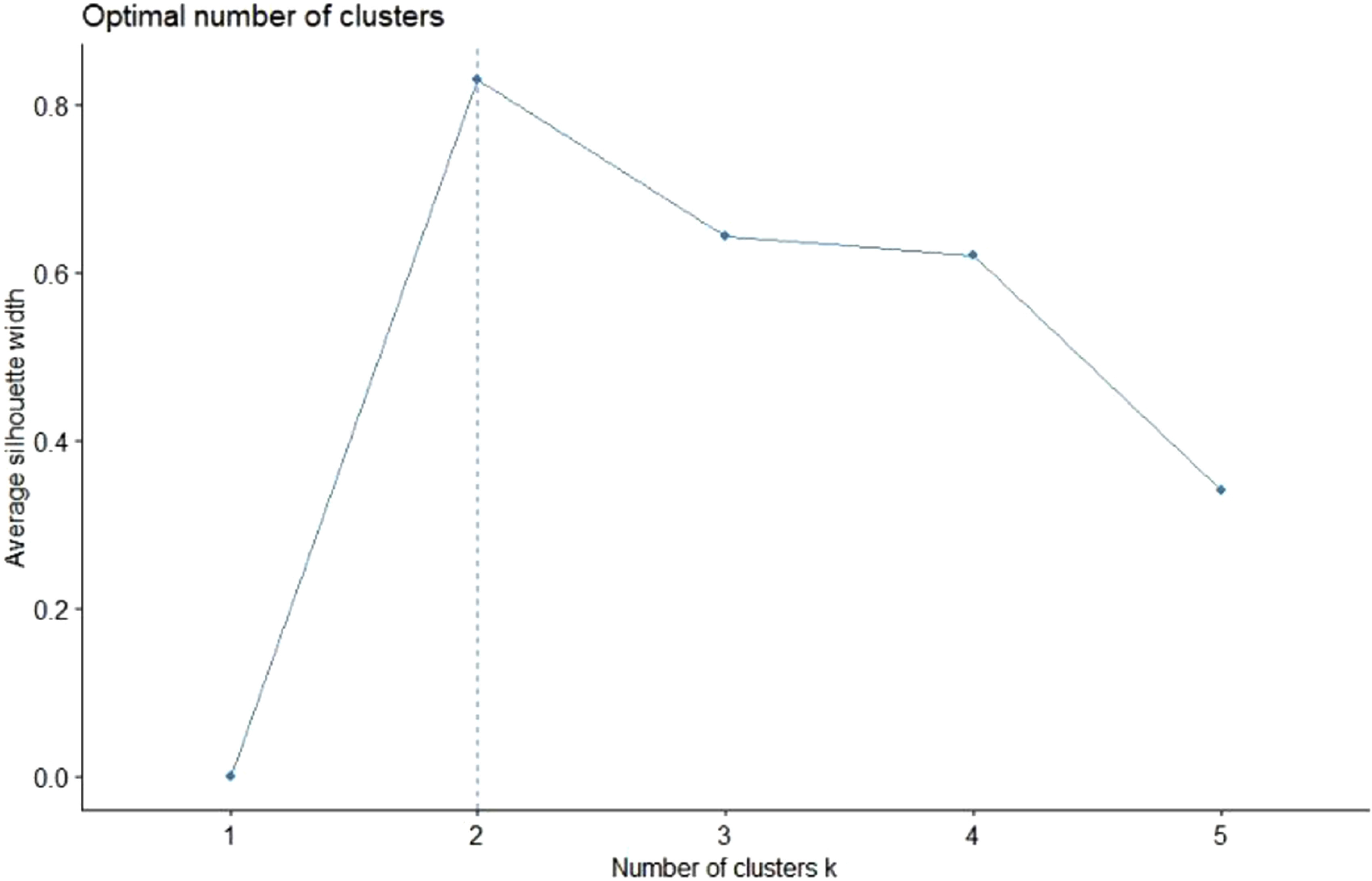
Average Silhouette Width for April 21 and 22, 2020.

To determine the optimal number of clusters, there are various methods, such as the elbow method and the average silhouette method. We prefer to use the average silhouette method, because it provides an objective estimate for the optimal number of clusters. Figure 2 shows the results of the average silhouette method for k-means clustering of the countries in terms of abs(%*Δβ*) and abs(%*Δγ*) for April 21 and 22, 2020.

The results show that 2 clusters maximize the average silhouette width, whereas using 3 clusters is the second optimal choice. Using 2 clusters seems to be a trivial option considering that Turkey stands out as a significant outlier, and the k-means algorithm will be forced to include Turkey in 1 cluster and all the other 9 countries in the other cluster. Therefore, we decided to use 3 clusters, which is also in line with our initial rough guess.

**FIGURE 3:**
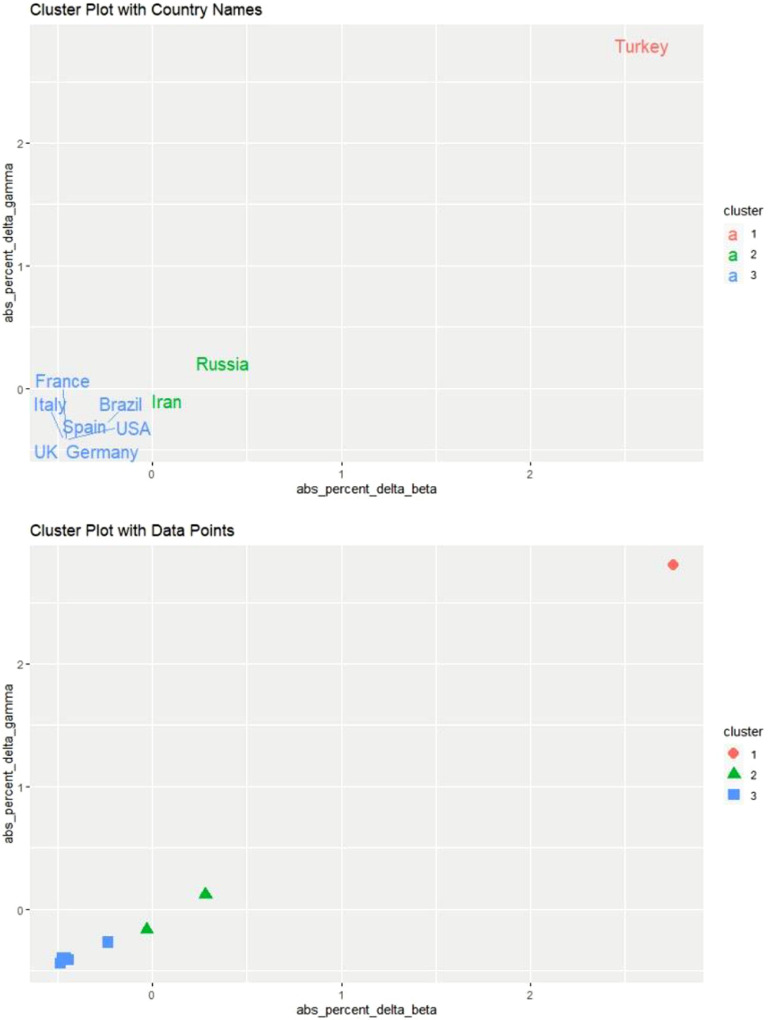
K-Means Cluster Analysis for April 21 and 22, 2020.

Figure 3 shows the results of our cluster analysis. We used 2 graphs, 1 with only country names and 1 with only data points, to provide a better visual representation.

The only difference between these results and our initial guess concerns Brazil. It turns out that Brazil is clustered with 6 developed countries, ie, France, Germany, Italy, Spain, the United Kingdom, and the United States. Yet, after carefully examining the second graph in Figure 3, it is evident that these developed countries stand closely grouped. In contrast, Brazil stands close to the border with the cluster of Iran and Russia.

These results clearly showed that obtaining robust parameter estimates is a bigger challenge in developing countries compared with developed countries. The higher gap between daily forecasts in developing countries can be attributed to potentially noisier data.

To explore the impact of time and more data on robustness, the model variation test is replicated with the same countries for May 19 and 20, 2020, and the results are presented in Table 2.

**TABLE 2:** *β* and *γ* Estimates With % Daily Change Between May 19 and 20, 2020

Country	*β* 05/19/2020	*β* 05/20/2020	abs(%*Δβ*)	*γ* 05/19/2020	*γ* 05/20/2020	abs(%*Δγ*)
Brazil	0.499	0.122	75.6%	0.420	0.054	87.1%
France	0.237	0.235	0.8%	0.097	0.096	1.0%
Germany	0.244	0.242	0.8%	0.099	0.098	1.0%
Iran	0.181	0.176	2.8%	0.094	0.092	2.1%
Italy	0.181	0.180	0.6%	0.081	0.081	0.0%
Russia	0.438	0.419	4.3%	0.325	0.307	5.5%
Spain	0.255	0.251	1.6%	0.105	0.099	5.7%
Turkey	0.217	0.215	0.9%	0.092	0.092	0.0%
United Kingdom	0.210	0.208	1.0%	0.108	0.108	0.0%
United States	0.203	0.200	1.5%	0.102	0.101	1.0%
Mean	0.267	0.225	9.0%	0.152	0.113	10.4%
Median	0.227	0.212	1.2%	0.101	0.097	1.0%

The results support *Hypothesis 2*. The parameter estimates become more robust as time progresses, particularly for developing countries. The apparent divergence between developing and developed countries in terms of robustness seems to have vanished with more data, except for Brazil. For countries other than Brazil, the absolute value of the percent daily change in parameter estimates ranges between 0.6% and 4.3% for *β* and 0.0% and 5.7% for *γ*. This time, Brazil stands out as an outlier with very high percent daily changes in both parameter estimates.

**FIGURE 4:**
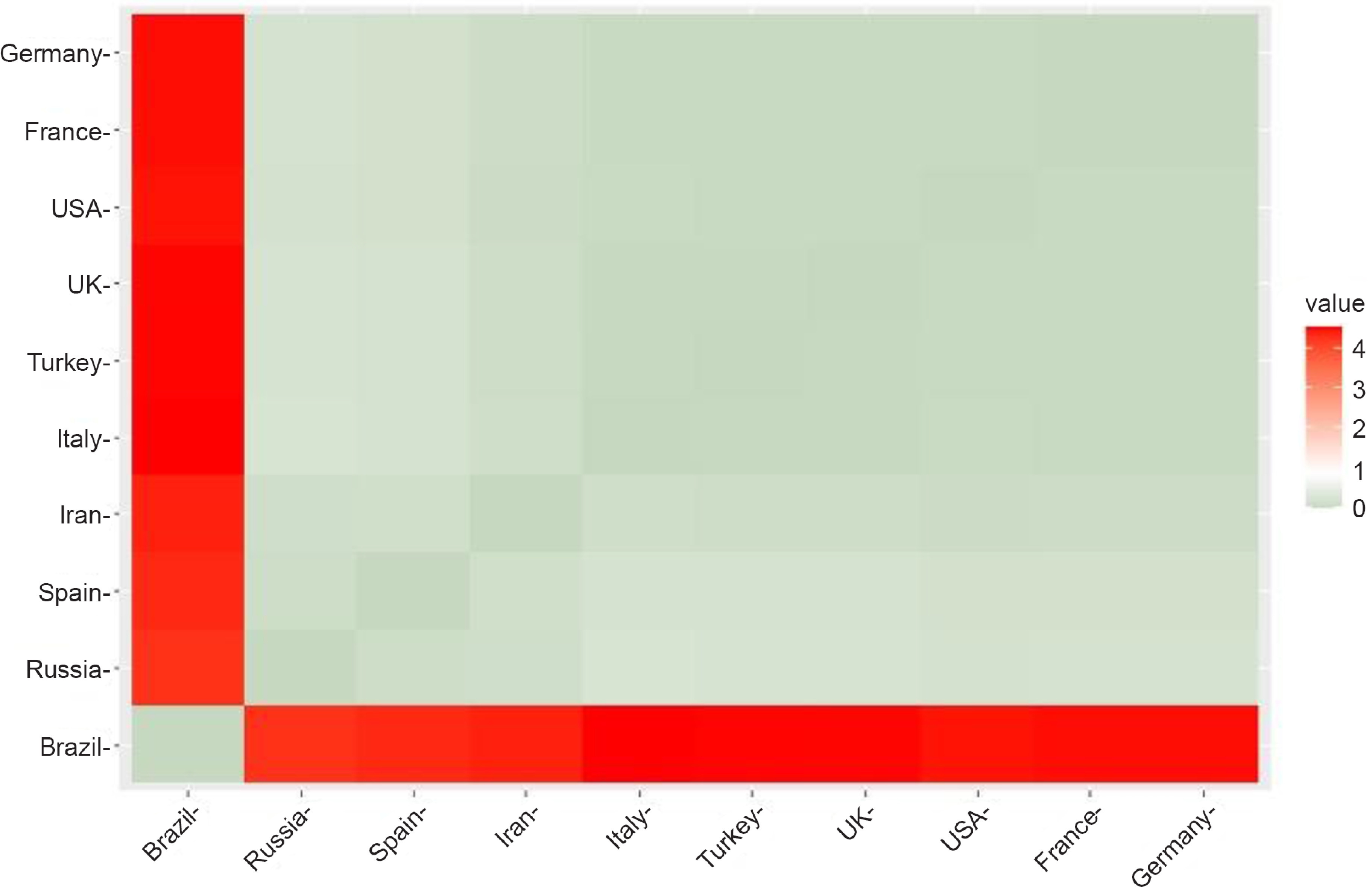
Distance Matrix Calculated From abs(%*Δβ*) and abs(%*Δγ*) for May 19 and 20, 2020.

Figure 4 shows a graphical representation of the distance matrix of countries calculated from abs(%*Δβ*) and abs(%*Δγ*) for May 19 and 20, 2020.

**FIGURE 5:**
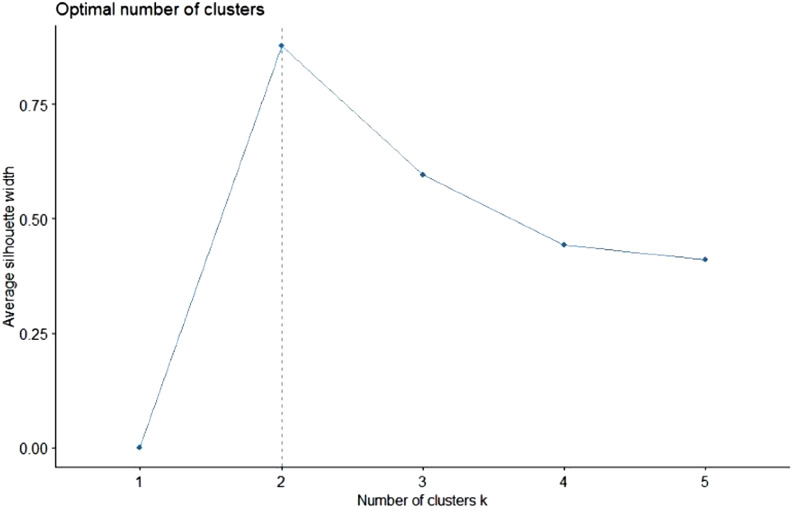
Average Silhouette Width for May 19 and 20, 2020.

Again, we used the k-means algorithm to perform a formal cluster analysis. Figure 5 shows the results of the average silhouette method for determining the optimal number of clusters.

Similar to our previous analysis for April 21 and 22, 2020, using 2 clusters seems to be the optimal choice, whereas the use of 3 clusters was the second-best option. However, this time, using 2 clusters can indeed be reasonable considering our observation that the results for all countries other than Brazil converge.

**FIGURE 6:**
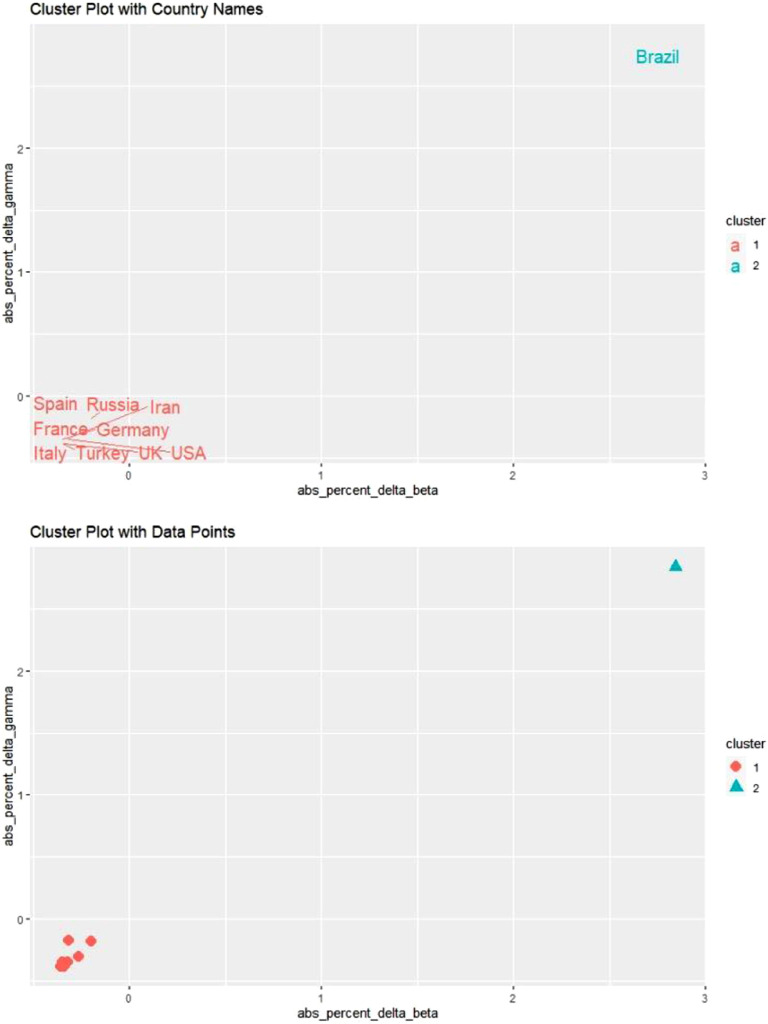
K-Means Cluster Analysis for May 19 and 20, 2020.

Figure 6 shows the results of our cluster analysis. As before, we used 2 graphs, 1 with only country names and 1 with only data points, to provide a better visual representation.

An examination of the second graph provides a visual proof that using 2 clusters was indeed the optimal choice. Because the marginal impact of each new data point on parameter estimates becomes smaller as time passes, the results were in line with our expectations. It is essential to point out that the impact of time on robustness was more significant for developing countries.

## INCOMPATIBILITY OF PARAMETER ESTIMATES WITH OBSERVED CHARACTERISTICS OF COVID-19

The recovery rate, *γ*, can be estimated as the reciprocal of the average number of days for the transition from I to R. For instance, a *γ* of 0.2 corresponds to 5 d for the infectious period. To this date, there is still no consensus in the medical community on the length of the contagious period for COVID-19.^25,26^

In this study, the median gamma estimate for COVID-19 was 0.187 on April 22, 2020, and 0.097 on May 20, 2020. These figures correspond to 5.3 d and 10.3 d for the infectious period, respectively. A recent study used 5 d for the infectious period of COVID-19.^27^ Another study argued that the infectious period seems longer for COVID-19 based on the few available clinical virological studies, perhaps lasting for 10 d or more after the incubation period.^25^ Hence, the median *γ* estimates can be deemed to be plausible.

On the other hand, *γ* estimates for Brazil, Turkey, and Iran on April 22, 2020, were 0.663, 0.743, and 1.422, respectively. These estimates correspond to a range of 0.7 to 1.5 d for the infectious period. Although the contagious period for COVID-19 is still deemed uncertain, this parameter range was unrealistic. These findings support *Hypothesis 4*. The model parameter estimates for some countries were not compatible with the actual disease dynamics. Hence, the models obtained at the end of this estimation procedure were unreliable.

Even with more data on May 20, 2020, the *γ* estimates for Brazil and Russia significantly diverged from the *γ* projections for other countries, which converge to a range of 0.08 to 0.11.

As a salient example, the SUTD did some research for the timing of the end of COVID-19 in different countries,^22^ using the same code from Batista,^10^ ie, the fitVirusCV19 function in MATLAB. The study achieved wide-spread instant popularity through news outlets all around the world, probably due to its optimistic predictions regarding the timing of the end of COVID-19.

For instance, for Turkey, the study predicted the date to reach 97% of the total expected cases as of May 16, 2020.^28^ Despite the favorable impact of preventive measures, the daily number of new cases in Turkey was still around 1000 (972 on May 20, 2020), while the pandemic was far from over. Considering the problems in parameter estimation, as mentioned earlier, particularly for developing countries such as Turkey, it was not surprising that the predictions turned out to be inaccurate and potentially misleading, both for the public and, more importantly, for policy- and decision-makers.

Furthermore, as Faranda et al. indicated, early estimates of COVID-19 show enormous fluctuations, despite the importance of having robust estimates of the time-asymptotic total number of infections.^18^ They showed that predictions are extremely sensitive to the reporting protocol and crucially depend on the last available data point before the maximum number of daily infections is reached.

SUTD, now, acknowledged that “model and data are inaccurate to the complex, evolving, and heterogeneous realities of different countries over time, and earlier predictions are no longer valid because the real-world scenarios have changed rapidly.” Thus, they removed the predictions from their website. They indicated that “the project is internalized,” and they referred visitors to other live public COVID-19 forecasting efforts around the world.^29^

## ROBUST ESTIMATION OF SIR MODEL

The curse of dimensionality states that the number of data points needed to estimate an arbitrary function with a given level of accuracy grows exponentially with the number of input variables (ie, dimensionality) of the function.^30^

For instance, an n-th order polynomial will achieve a perfect fit for n+1 data points. However, such a model will seriously lack the ability to generalize, and it will not be able to generate accurate predictions. Instead, a simple linear regression will be much superior in terms of predictive performance and the ability to generalize over unseen data.

The presence of noise exacerbates the problem, and the real-world data are inherently noisy. The data for COVID-19 are imperfect and incomplete. This finding is even more so for developing and underdeveloped countries. Most developing countries suffer from an acute lack of COVID-19 testing capacity, and they either collect low-quality data or do not record deaths at all.^31^

**FIGURE 7:**
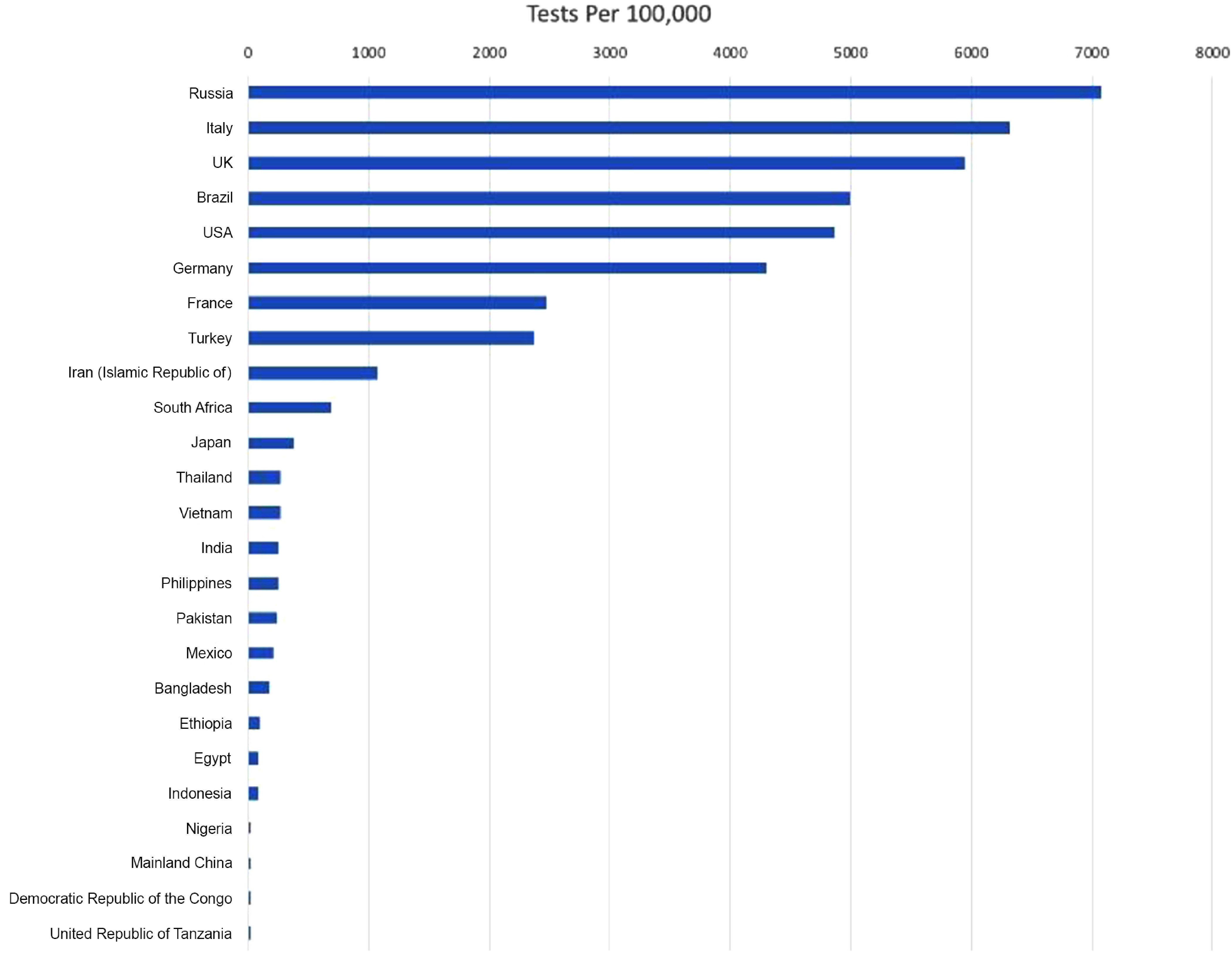
Tests per 100,000 for the Top 25 Most Populous Countries as of May 30, 2020.

Figure 7 depicts the number of tests per 100,000 for the top 25 most populous countries as of May 30, 2020.^23^

As can be seen from the figure, the number of tests per 100,000 for Ethiopia, Egypt, Indonesia, Nigeria, Mainland China, Democratic Republic of Congo, and United Republic of Tanzania is below 100, which suggests a serious lack of COVID-19 testing capacity for some of the most populous countries in the world.

In addition, death tolls are sporadically revised in many countries, which casts further doubt on the reported figures.^32-34^ This inevitably makes the COVID-19 data highly noisy, especially for developing countries. Even for developed countries, such as the United States and Italy, there is new research that shows that coronavirus deaths could be up to double the official counts.^35^ More complex models tend to learn the noise as well as signal, which is not intended.

This phenomenon is closely related to the principle of “Occam’s razor”,^36^ ie, “*pluralitas non est ponenda sine necessitate”* or “plurality should not be posited without necessity.” In other words, “of two competing theories, the simpler explanation of an entity is to be preferred.”

Therefore, especially in the initial phase of the pandemic with insufficient data, we propose to estimate only *β* instead of trying to estimate *β* and *γ*, simultaneously. The infection rate, *β*, is dependent on many factors, such as population density,^37^ demographics,^38^ and social distancing measures.^39^ On the other hand, the removal rate, *γ*, is the reciprocal of the infectious period, which is expected to be more stable compared with *β*. Hence, we prefer to take *γ* from the literature and estimate *β* only. As demonstrated below, this effectively overcomes the problem of estimating robust parameters for the basic SIR model, particularly for noisier data from developing countries. It also eliminates the problem of incompatibility between parameter estimates and actual disease characteristics.

Because the code provided by Batista^10^ estimates *β* and *γ*, simultaneously, we modified the code to allow for single parameter estimation.

First, we repeat the model variation test for April 21 and 22, 2020, with *γ* set equal to 0.2 by using the modified code to estimate the remaining parameter, *β*. A *γ* of 0.2 corresponds to 5 d for the infectious period of COVID-19,^27^ The estimate of *β* and the absolute value of the percent daily changes in parameter estimates are presented in Table 3 for April 21 and 22, 2020.

**TABLE 3:** *β* Estimates With % Daily Change for Fixed *γ* Between April 21 and 22, 2020

Country	*β* 04/21/2020	*β* 04/22/2020	abs(%*Δβ*)	*γ* 04/21/2020	*γ* 04/22/2020	abs(%*Δγ*)
Brazil	0.337	0.336	0.3%	0.200	0.200	0.0%
France	0.364	0.350	3.9%	0.200	0.200	0.0%
Germany	0.357	0.355	0.4%	0.200	0.200	0.0%
Iran	0.313	0.319	1.8%	0.200	0.200	0.0%
Italy	0.316	0.314	0.5%	0.200	0.200	0.0%
Russia	0.382	0.378	0.9%	0.200	0.200	0.0%
Spain	0.366	0.379	3.4%	0.200	0.200	0.0%
Turkey	0.374	0.370	1.1%	0.200	0.200	0.0%
United Kingdom	0.359	0.357	0.6%	0.200	0.200	0.0%
United States	0.370	0.368	0.6%	0.200	0.200	0.0%
Mean	0.354	0.353	1.4%	0.200	0.200	0.0%
Median	0.362	0.356	0.7%	0.200	0.200	0.0%

Compared with the results in Table 1, the new results obtained by estimating *β* only were evidently more robust. The absolute value of the percent daily change in *β* estimate ranges between 0.3% and 3.9%, with a mean of 1.4%. On the other hand, the same measure in the previous version, where both parameters were estimated simultaneously, ranged between 0.6% and 174.0%, with a mean of 26.5%.

Next, we perform a structured permutation test by means of perturbing *γ* by ±10% for April 21, 2020. The results are presented in Tables 4 and 5.

**TABLE 4:** *β* Estimates for *γ* = 0.20 and *γ* = 0.22 on April 21, 2020

Country	*β* 04/21/2020	*β* 04/21/2020	abs(%*Δβ*)	*γ* 04/21/2020	*γ* 04/21/2020	abs(%*Δγ*)
Brazil	0.337	0.357	5.7%	0.200	0.220	10.0%
France	0.364	0.384	5.5%	0.200	0.220	10.0%
Germany	0.357	0.397	11.3%	0.200	0.220	10.0%
Iran	0.313	0.333	6.3%	0.200	0.220	10.0%
Italy	0.316	0.335	6.2%	0.200	0.220	10.0%
Russia	0.382	0.402	5.2%	0.200	0.220	10.0%
Spain	0.366	0.402	9.8%	0.200	0.220	10.0%
Turkey	0.374	0.394	5.3%	0.200	0.220	10.0%
United Kingdom	0.359	0.379	5.6%	0.200	0.220	10.0%
United States	0.370	0.392	6.2%	0.200	0.220	10.0%
Mean	0.354	0.377	6.7%	0.200	0.220	10.0%
Median	0.362	0.388	5.9%	0.200	0.220	10.0%

**TABLE 5:** *β* Estimates for *γ* = 0.20 and *γ* = 0.18 on April 21, 2020

Country	*β* 04/21/2020	*β* 04/21/2020	abs(%*Δβ*)	*γ* 04/21/2020	*γ* 04/21/2020	abs(%*Δγ*)
Brazil	0.337	0.318	5.9%	0.200	0.180	10.0%
France	0.364	0.344	5.5%	0.200	0.180	10.0%
Germany	0.357	0.357	0.1%	0.200	0.180	10.0%
Iran	0.313	0.293	6.3%	0.200	0.180	10.0%
Italy	0.316	0.316	0.0%	0.200	0.180	10.0%
Russia	0.382	0.344	10.0%	0.200	0.180	10.0%
Spain	0.366	0.362	1.2%	0.200	0.180	10.0%
Turkey	0.374	0.355	5.3%	0.200	0.180	10.0%
United Kingdom	0.359	0.339	5.6%	0.200	0.180	10.0%
United States	0.370	0.352	4.7%	0.200	0.180	10.0%
Mean	0.354	0.338	4.5%	0.200	0.180	10.0%
Median	0.362	0.344	5.4%	0.200	0.180	10.0%

When *γ* increases by 10%, the absolute value of the percent change in *β* estimate ranges between 5.2% and 11.3%, with a mean of 6.7%. On the other hand, when *γ* decreases by 10%, the absolute value of the percent change in *β* estimate ranges between 0.0% and 10.0%, with a mean of 4.5%. Hence, the results of the structured permutation test also validate the robustness of the SPE approach.

In addition, the incompatibility of parameter estimates with actual disease characteristics is also resolved by this new approach. As *γ* is set equal to a figure taken from the literature, *β* remains as the only potential source of incompatibility. Yet, the resulting *β* estimates range in a relatively tight and plausible interval of 0.313 and 0.382, with a mean of 0.354 for *γ* = 0.2.

## AN ILLUSTRATIVE EXAMPLE FROM NORWAY AND NORWEGIAN COUNTIES

Norway was one of the countries that implemented tough restrictions to follow the containment strategy toward the COVID-19 pandemic. Following WHO’s declaration of the pandemic, the announced measures involved emergency shutdowns of many public and private institutions, including schools and kindergartens. The country managed to bring down the effective reproduction number, R*_e_*, to 0.7 by early April.^40^ It was also among the countries that provided open access data at the county-level.

**FIGURE 8:**
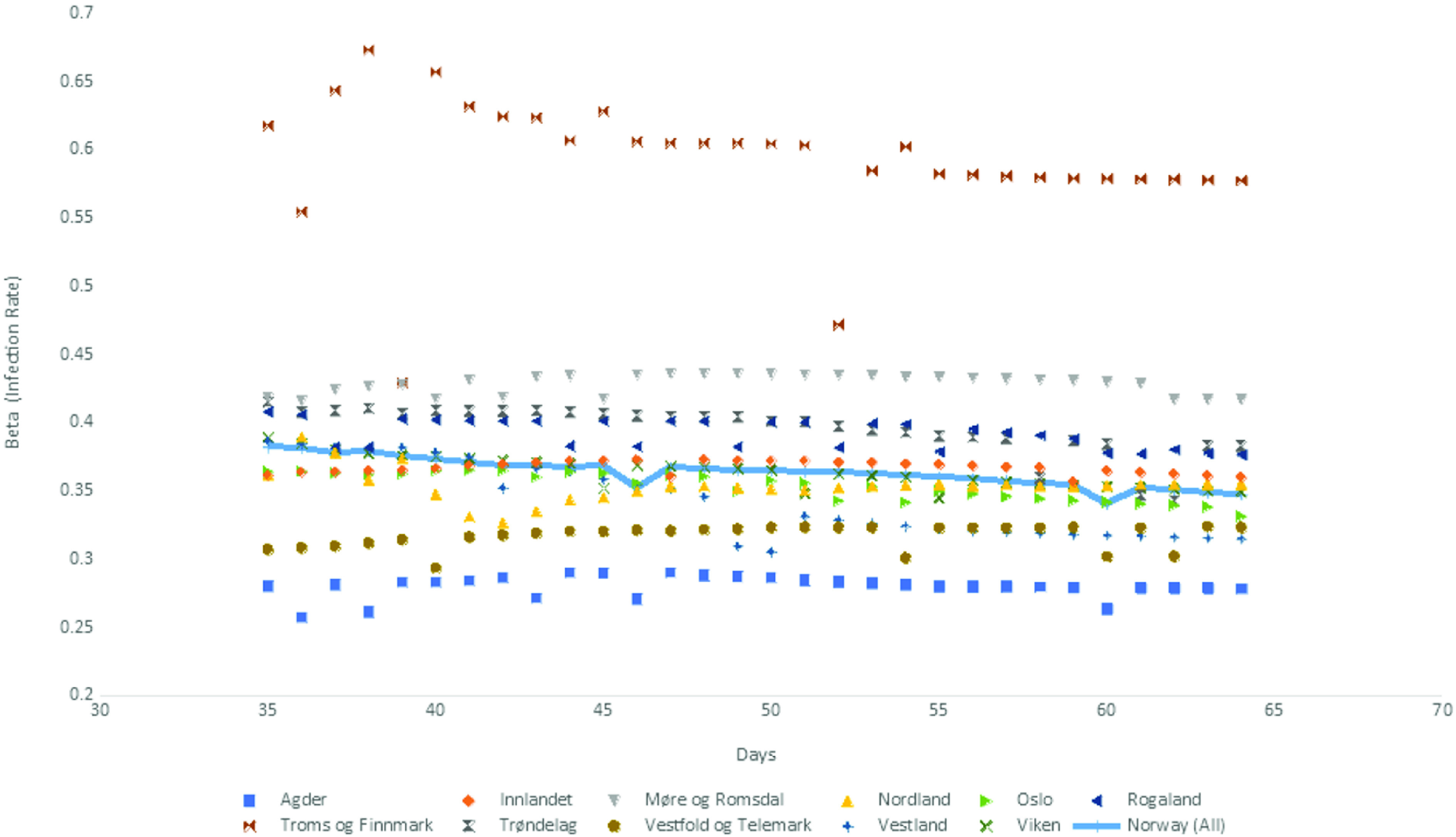
*β* (Infection Rate) for Norway and Counties in Norway.

**FIGURE 9:**
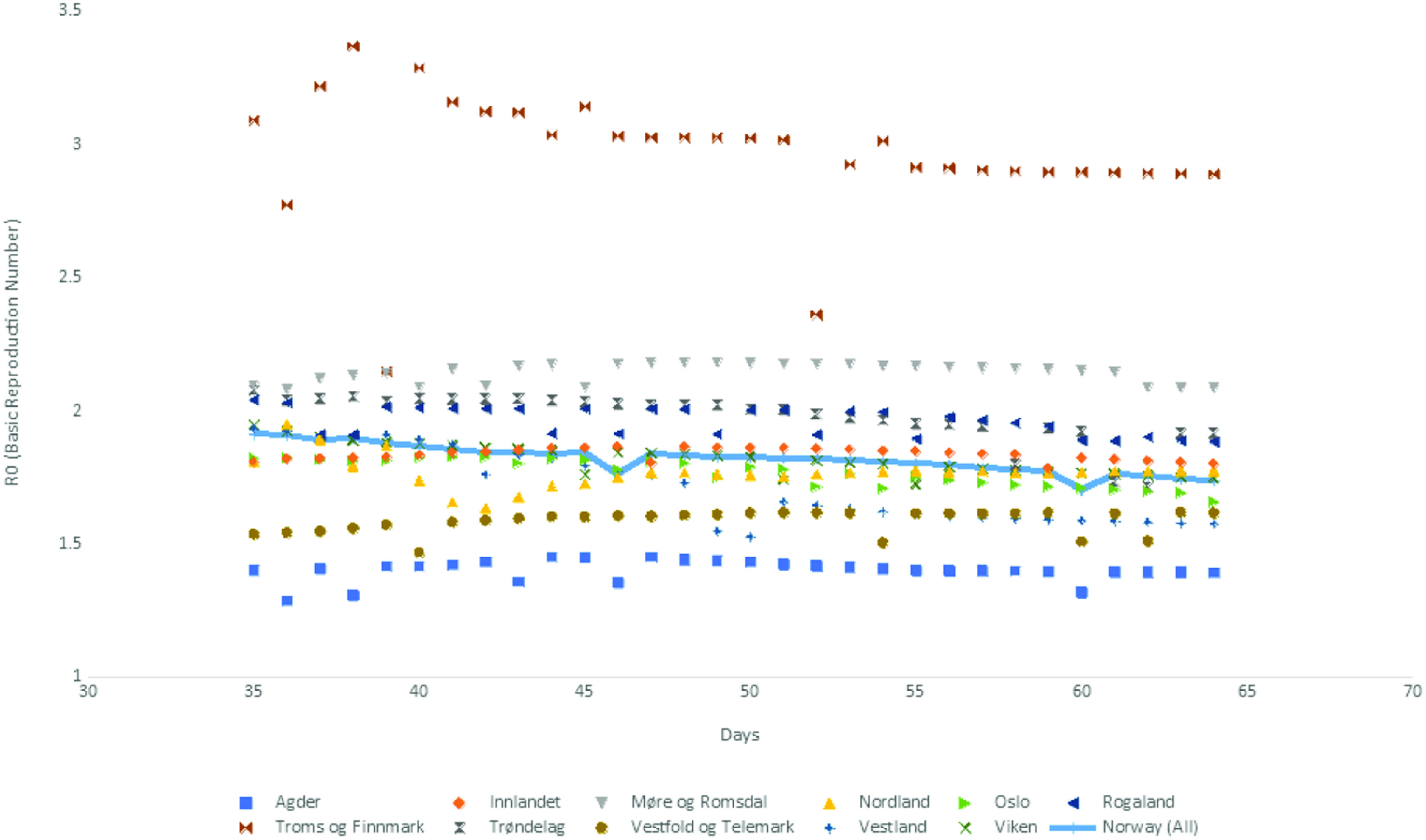
*R*_0_ (Basic Reproduction Number) for Norway and Counties in Norway.

**FIGURE 10:**
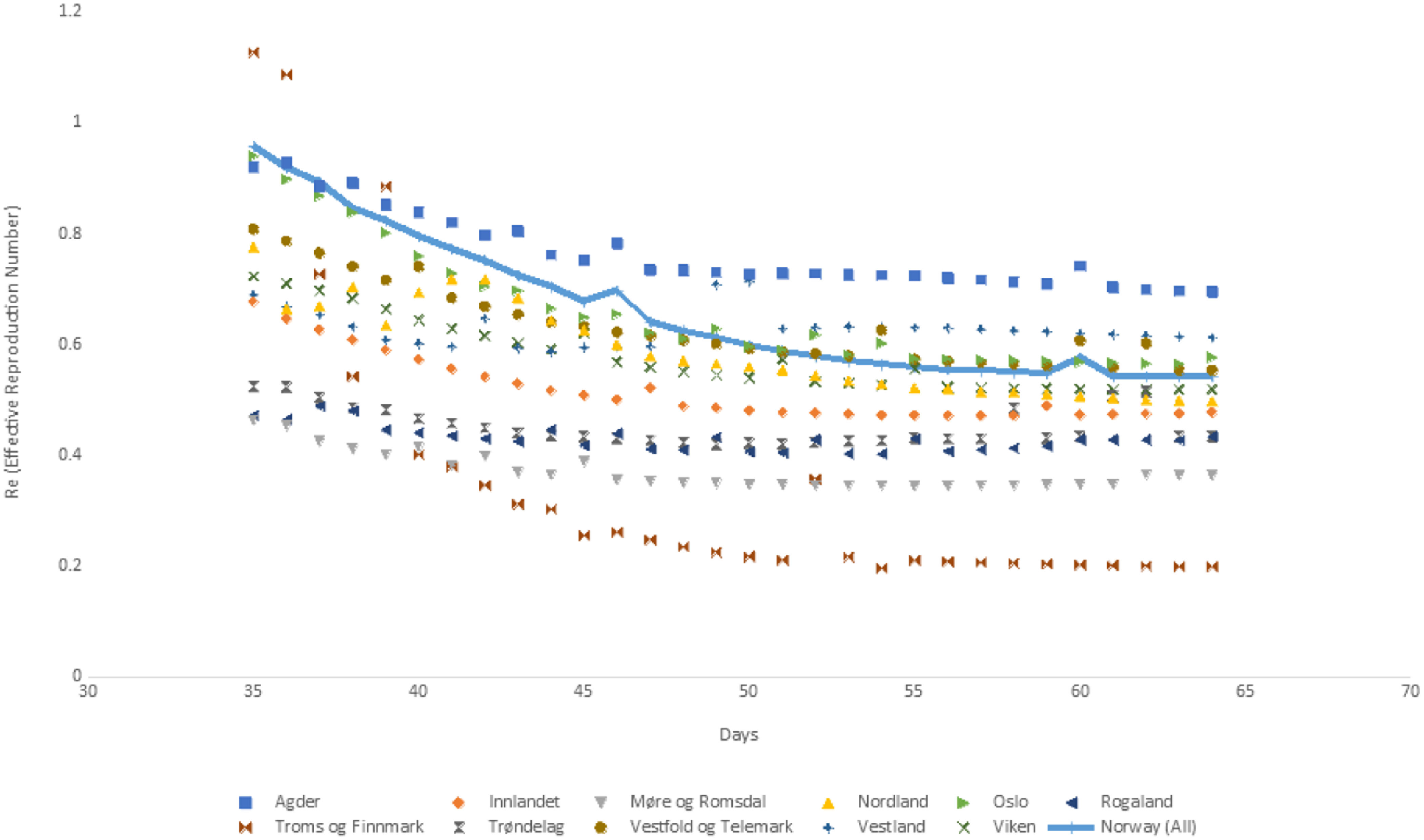
*R_e_* (Effective Reproduction Number) for Norway and Counties in Norway.

We used Norwegian data to test our proposed SPE approach both at the country and county levels. *γ* is set equal to 0.2, corresponding to 5 d for the infectious period, which is taken from a report published by the Norwegian Institute of Public Health.^27^ We obtained a time-series of the infection rate, *β*, the basic reproduction number, R_0_, and the effective reproduction number, R*_e_*, for the 11 counties and the whole country. The time series covered a 1-mo period, which was between the day 35 and 64 of the pandemic. Figures 8, 9, and 10 depict these time series, whereas the time series for R*_e_* is also tabulated in Table 6.

**TABLE 6:** *R_e_* (Effective Reproduction Number) for Norway and Counties in Norway

Days	Agder	Innlandet	More_og_Romsdal	Nordland	Oslo	Rogaland	Troms_og_Finnmark	Trondelag	Vestfold_og_Telemark	Vestland	Viken	NorwayAll
**35**	0.92	0.68	0.46	0.78	0.94	0.47	1.13	0.53	0.81	0.69	0.72	0.96
**36**	0.93	0.65	0.45	0.67	0.90	0.47	1.09	0.52	0.79	0.67	0.71	0.92
**37**	0.89	0.63	0.43	0.67	0.87	0.49	0.73	0.51	0.77	0.65	0.70	0.89
**38**	0.89	0.61	0.41	0.71	0.84	0.48	0.54	0.49	0.74	0.63	0.68	0.84
**39**	0.85	0.59	0.40	0.64	0.80	0.45	0.89	0.48	0.72	0.61	0.67	0.82
**40**	0.84	0.57	0.41	0.69	0.76	0.44	0.40	0.47	0.74	0.60	0.64	0.80
**41**	0.82	0.56	0.38	0.72	0.73	0.44	0.38	0.46	0.68	0.60	0.63	0.77
**42**	0.80	0.54	0.40	0.72	0.71	0.43	0.35	0.45	0.67	0.65	0.62	0.75
**43**	0.80	0.53	0.37	0.69	0.70	0.43	0.31	0.44	0.65	0.59	0.60	0.73
**44**	0.76	0.52	0.36	0.64	0.67	0.45	0.30	0.44	0.64	0.59	0.59	0.70
**45**	0.75	0.51	0.39	0.63	0.65	0.42	0.26	0.43	0.63	0.60	0.62	0.68
**46**	0.78	0.50	0.36	0.60	0.66	0.44	0.26	0.43	0.62	0.60	0.57	0.70
**47**	0.74	0.52	0.35	0.58	0.62	0.41	0.25	0.43	0.62	0.60	0.56	0.64
**48**	0.73	0.49	0.35	0.57	0.61	0.41	0.24	0.42	0.61	0.61	0.55	0.63
**49**	0.73	0.49	0.35	0.57	0.63	0.43	0.23	0.42	0.60	0.71	0.55	0.61
**50**	0.73	0.48	0.35	0.56	0.60	0.41	0.22	0.42	0.59	0.71	0.54	0.60
**51**	0.73	0.48	0.35	0.56	0.59	0.41	0.21	0.42	0.59	0.63	0.57	0.59
**52**	0.73	0.48	0.35	0.55	0.62	0.43	0.36	0.42	0.58	0.63	0.53	0.58
**53**	0.73	0.47	0.35	0.54	0.58	0.40	0.22	0.43	0.58	0.63	0.53	0.57
**54**	0.73	0.47	0.35	0.53	0.60	0.40	0.20	0.43	0.63	0.63	0.53	0.56
**55**	0.73	0.47	0.35	0.52	0.57	0.43	0.21	0.43	0.57	0.63	0.56	0.56
**56**	0.72	0.47	0.35	0.52	0.57	0.41	0.21	0.43	0.57	0.63	0.52	0.56
**57**	0.72	0.47	0.35	0.52	0.57	0.41	0.21	0.43	0.57	0.63	0.52	0.55
**58**	0.71	0.47	0.35	0.52	0.57	0.41	0.21	0.49	0.57	0.63	0.52	0.55
**59**	0.71	0.49	0.35	0.51	0.57	0.42	0.20	0.43	0.56	0.62	0.52	0.55
**60**	0.74	0.47	0.35	0.51	0.57	0.43	0.20	0.44	0.61	0.62	0.52	0.58
**61**	0.70	0.47	0.35	0.51	0.57	0.43	0.20	0.51	0.56	0.62	0.52	0.54
**62**	0.70	0.48	0.37	0.50	0.57	0.43	0.20	0.52	0.60	0.62	0.52	0.54
**63**	0.70	0.48	0.37	0.50	0.57	0.43	0.20	0.43	0.55	0.62	0.52	0.54
**64**	0.70	0.48	0.36	0.50	0.58	0.43	0.20	0.43	0.55	0.61	0.52	0.54

An examination of Figures 8 and 9 provides a visual proof that robust estimates of *β* and R_0_ are obtained for all the counties in Norway, with the possible exception of Troms og Finnmark. This is probably due to data collection problems in that particular county because the data for all the other counties and the whole country generated robust parameter estimates.

If R*_e_* is above 1.0, then the number of infected people grows exponentially. Hence, the threshold level for R*_e_* that can be deemed safe should be less than or equal to 1.0. Countries, such as Germany and Czechia, have declared this threshold level to be 1.0 to start easing restrictions and preventive measures.^41,42^

Norway, on the other hand, waited until R*_e_* came down to 0.7, to even consider easing. Bent Hoeie, the Norwegian Minister of Health and Care Services, announced that R*_e_* was equal to 0.7 as of April 6, 2020.^40^ This date corresponded to day 46 of the pandemic. An examination of Table 6 shows that our R*_e_* estimate for day 46 is indeed 0.70, which is congruent with the estimate made by the Norwegian health authorities.

Figure 10 and Table 6 show that almost half of the counties in Norway were already in the safe zone in terms of R*_e_* at the beginning of the 1-mo period, ie, day 35 of the pandemic. Agder, Nordland, Oslo, Troms og Finnmark, Vestfold og Telemark, and Viken had R*_e_* values higher than 0.7. R*_e_* values for all these counties quickly came down to 0.7 in a few days with the exception of Agder, which reached the threshold level on day 61 of the pandemic.

Norway did not start easing the restrictions until April 13, 2020, ie, day 53 of the pandemic.^43^ The easing has been slow and gradual.

## CONCLUDING REMARKS

Predicting the progress of COVID-19 is a crucial problem for policy- and decision-makers. However, the models used for this purpose are prone to significant estimation errors. Therefore, the results obtained from these models should be viewed with extreme caution.

We do not claim that our proposed SPE approach makes the basic SIR model an optimal tool for predicting the progress of COVID-19 or any other pandemic. If data permit, more sophisticated models should be preferred. The SPE can be a useful approach to obtain robust parameter estimates if the basic SIR is the model of choice. In fact, the SPE approach is nothing more than an application of one of the most critical tenets of data science, ie, the predilection for simpler models if data are limited and noisy. Hence, the same principle can be applied to any model with any number of parameters. For instance, if a model requires the estimation of 3 parameters, fixing 1 parameter and estimating the other 2 is going to be more robust than the simultaneous estimation of all 3 parameters.

From a policy perspective, monitoring the current state of the pandemic is at least as important as predicting its progress. A fundamental policy question persists regarding the timing for easing and eventually lifting limitations such as lockdowns. Estimating the instantaneous reproduction number by using a rather robust method such as Bayesian statistical inference can shed light on the optimal timing for easing limitations.^44,45^
